# Effect of high-dose N-acetyl cysteine on the clinical outcome of patients with diabetic peripheral neuropathy: a randomized controlled study

**DOI:** 10.1186/s13098-025-01624-9

**Published:** 2025-03-04

**Authors:** Sherien Mohamed Emara, Sarah Farid Fahmy, Mona Mohamed AbdelSalam, Lamia Mohamed El Wakeel

**Affiliations:** 1 Clinical Pharmacy Department, Faculty of Pharmacy, British University, Cairo, Egypt; 2 Clinical Pharmacy Department, Faculty of Pharmacy, Ain Shams University, Cairo, Egypt; 3 Department of Endocrinology, Faculty of Medicine, Ain Shams University Hospitals, Cairo, Egypt

**Keywords:** Diabetic peripheral neuropathy, N-acetyl cysteine, Glutathione peroxidase, Nuclear factor erythroid-2 related factor, Tumor necrosis factor

## Abstract

**Background:**

Diabetic peripheral neuropathy (DPN) is a vastly common and bothersome disorder with a clinically challenging course of treatment affecting patients with diabetes. This study aimed to evaluate the efficacy and safety of high dose oral N-acetyl cysteine (NAC) as adjuvant therapy on clinical outcome of DPN.

**Methods:**

A prospective, randomized, parallel, open label, controlled clinical trial. Ninety eligible DPN patients were randomly assigned to either *control group* receiving standard of care or *NAC group* receiving standard of care treatment and NAC at a dose of 2400 mg/day for 12 weeks. Glutathione peroxidase (GPx), nuclear factor erythoid-2 related factor (NRF-2) and tumor necrosis factor (TNF) were measured at baseline and after 12 weeks to assess anti-oxidant and anti-inflammatory properties. Michigan neuropathy screening instrument (MNSI), Toronto clinical neuropathy score (TCNS), Diabetic neuropathy score (DNS), Diabetes-39 quality of life questionnaire (DQOL) and pain score were assessed at baseline and after 12 weeks.

**Results:**

NAC group showed a significant increase (p < 0.05) in NRF-2 by 25.3% and GPx by 100% and a decline of 21.45% in TNF-alpha levels versus controls that reported a decline in NRF-2 and GPx and an increase in TNF-alpha. HgbA1C and AST levels significantly decreased in NAC versus controls (7.2 ± 1 vs 8 ± 1.1, p = 0.028 and 29.1 vs 55.4, p = 0.012) respectively. NAC administration resulted in a significant decline in MNSA, TCNS, DNS and pain scores versus controls that showed increase in all scores. The QOL total score and the anxiety and energy and mobility domain scores significantly decreased in the NAC group versus controls, p < 0.001.

**Conclusion:**

High dose NAC administered for 12 weeks modulated inflammation by reducing TNF-alpha and increasing GPx and NRF2 versus controls. NAC improved clinical outcomes of DPN reflected by a decline in neuropathy and pain scores and an improvement in QOL.

**Clinical trial registration number:**

NCT04766450

## Background

Diabetic peripheral neuropathy (DPN) is the most frequent long-term microvascular consequence of diabetes mellitus (DM) encompassing a wide spectrum of clinical and subclinical symptoms that are linked to loss of peripheral nerve fibers [1]. It is one of the most challenging diabetes complications to treat [2].

In DM patients, the incidence rate of neuropathy rises up to 30% and up to 50% of DM patients develop neuropathy during their lifetime [3]. In Egypt, it was confirmed that 29.3% of Egyptian diabetic patients suffer from peripheral neuropathy [4]. DPN is more common in DM type 2 than those with type 1 and in women than men [4, 5].

DPN can cause sensory, motor, or autonomic symptoms [6]. Pain is typically coupled with sensory sensations [4], with severe pain more pronounced in females and connected with the severity of DPN [5]. It is also a complicated and multi-dimensional illness that impairs psychosocial functioning resulting in pain-related impairment due to elevated levels of anxiety and depression [7].

DPN is a significant factor in the development of foot ulceration, which can lead to foot amputation [8]. One of the main reasons why older patients fear of falling and kinesiophobia is the loss of sensation that may lead to impairment in their quality of life [7, 9].

The only FDA approved agents for treatments of diabetic neuropathy are gabapentin, pregabalin, and duloxetine. The discovery of novel medications for the treatment of diabetic neuropathy is warranted due to the emergence of drug tolerance, possible toxicity, and insufficient alleviation [2]. DPN has a significant health burden, yet it’s best course of treatment has not been elucidated and remains clinically challenging due to its complex underlying pathogenesis [1].

A wide spectrum of pathogenetic mechanisms underly DPN with oxidative stress, inflammation with the release of proinflammatory cytokines and mitochondrial dysfunction being the major contributors [10].

Alpha-lipoic acid (ALA), vitamin E, and acetyl-L-carnitine have all been studied in patients with DPN over the past ten years with variable results pertaining to their different antioxidant and anti-inflammatory effects on DPN [8, 12–14]

Additionally, in preclinical models of diabetic neuropathy, natural supplements including curcumin, resveratrol, and melatonin as well as other antioxidants (Vitamins A, C, E and ALA) that target mitochondrial dysfunction have showed promising results [15–18]*N-acetylcysteine (NAC)* was previously shown to impact multiple pathogenetic pathways in DPN, making it a potential candidate for treatment. NAC is a GSH precursor and a cysteine prodrug with well-known anti-inflammatory and anti-oxidant properties [19, 20]. NAC has potent anti-inflammatory benefits mediated by preventing NF-B activation and decreasing the release of cytokines [21]. Moreover, studies have reported that NAC induces analgesia via suppression of matrix metalloproteinases and inhibition of nociceptive responses [1, 22–24].

Hence, considering the multiple pathogenetic pathways of diabetic neuropathy, including oxidative stress and inflammation, NAC use might be potentially useful for managing DPN.

Two Previous studies [1, 25] addressing NAC use were limited to only modest doses of NAC (600–1200 mg/day) and short duration of treatment (8 weeks) and showed a 32% significant improvement in glutathione peroxidase levels and a 6% improvement in pain score, with a recommendation of the use of higher doses of NAC for longer duration.

To the best of our knowledge, there are no clinical studies to date exploring the influence of high dose oral NAC on the management of PDN.

## Methods

### Aim

The current study aimed to evaluate the efficacy and safety of high dose oral NAC (2400 mg/day) as an adjunct therapy on oxidative stress, inflammatory markers and clinical outcomes of patients with type 2 diabetes suffering from diabetic peripheral neuropathy.

### Design

A prospective, randomized, parallel, open label, controlled clinical trial

### Setting

The outpatient Endocrinology clinic, Department of Endocrinology, Faculty of Medicine, Ain Shams University, Cairo, Egypt.

### Ethical consideration

The study protocol was approved by the research ethics committee of the faculty of Pharmacy Ain shams university approval number; (RHDIRB2020110301 REC #15). The study was conducted in accordance to the regulations of the declaration of Helsinki as revised in 2013. The study was registered at www.clinicaltrial.gov (Registration Number: NCT04766450). All recruited patients were required to fill a written informed consent

### Patients

All patients presenting to the department were screened for eligibility according to the following inclusion/exclusion criteria. Included patients were male and females aged from 35–55 years and BMI (25- greater than 30 kg/m^2^) with Type 2 diabetes and a HbA1c of (6%-9%) together with a confirmed diagnosis of Diabetic Neuropathy (by pinprick, temperature probe, ankle reflex, and vibration perception (128-Hz tuning fork) or pressure sensation (10 g monofilament test).

Exclusion criteria included any of the following; those with acute and/or chronic inflammatory conditions, malignancy, receiving any antioxidants or anti-inflammatory medicines, pregnancy, lactation or expecting to get pregnant during the study period, receiving medical, psychological, or any pharmacological interventions that might interfere with data collection or interpretation of study data and hypersensitivity to N-acetylcysteine.

Patients were to be excluded after enrollment if they were non-adherent to the treatment (using the medication for less than 80% of study period) or refused or decided to withdraw from the study.

Eligible patients were randomly assigned using a research randomizer application (Study Randomizer) to one of the two groups, Control or NAC group. Control patients (n = 28) received standard of care treatment only, while NAC patients (n = 28) received the standard of care treatment plus N-acetyl cysteine. NAC was given in the form of sachets (supplied by Xeedia Pharma) at a dose of 1200 mg twice daily [26].

#### Medication administration and follow up

The standard of care adopted at our institution was comprised of anti-diabetic medications and vitamin B complex.

Adherence to treatment (NAC) was assessed by the clinical pharmacist and determined by handing empty sachets and counting sachets left in the box at the end of the month and patients were considered adherent to treatment if at least 80% of all doses were taken.

Adherence to standard of care medications was assessed in both groups by following up with patients regularly and patient self-reporting in a pre-designed card for missed doses of medications.

Both groups were followed up for a period of three months regularly every other week and weekly by phone for monitoring the occurrence of any adverse drug reactions.

### Baseline assessment

At baseline, all patients were subjected to a full data collection by the clinical pharmacist and the clinical examination was performed by the assigned physician together with laboratory evaluation. Data collection included; demographics and anthropometric data (age, gender, weight, height, waist circumference), medical history which included: past medical history, duration of diabetes, duration of diabetic peripheral neuropathy, medication history which included: anti-diabetic medications, peripheral neuropathy medications, any other past and current prescribed and OTC medications.

At baseline all patients were educated about the disease and NAC role together with the expected side effects and how to report any side effects using a pre-designed side effect reporting card.

### Clinical examination

Was performed at baseline and at the end of the study using several tools including, the Michigan Neuropathy screening instrument (MNSI), the Toronto clinical scoring system (TCNS) and the diabetic neuropathy symptom score (DNS). Pain assessment was done using the Numeric Pain rating scale (NPRS).

The validated and translated Arabic version of *the Michigan Neuropathy screening instrument* (MNSI) was used which is a questionnaire with 15 questions that evaluate both positive (pain, temperature sensation, tingling) and negative (numbness) sensory symptoms, cramps and muscle weakness, foots ulcers or cracks and amputation. Neuropathy was defined as seven or more positive responses on the MNSI questionnaire [27].

The *Toronto clinical scoring system* consists of three parts: symptom scores, reflex scores and sensory test scores. The maximum score is 19, zero to five points indicate no DPN; six to eight points indicate mild DPN; nine to 11 points indicate moderate DPN; and 12 to 19 points, indicate severe DPN [27].

The *Diabetic Neuropathy symptom score* consists of 4 questions assessing pain, numbness, tingling and ataxia. The maximum score of the DNS is four points, one point or more indicates neurological abnormalities [28].

The *Numeric Pain rating scale* was performed by asking patients to circle the number between 0 and 10 representing their degree of pain [29].

### Quality of life

Assessment was done using the diabetes-39 (D-39) quality of life questionnaire which evaluates several aspects of patients ‘condition namely; Energy and mobility (15 items), diabetes control (12 items), anxiety and worry (4 items), social overload (5 items), and sexual behavior (3 items). These five components of the diabetes-39 (D-39) instrument are used to evaluate the QOL of people with type 2 diabetes. As the total score increases, it indicates the impaired levels of QOL [1, 30, 31] together with classifying and staging of DPN [27].

### Laboratory evaluation

Was done at baseline and after 3 months and included evaluation of:

***Inflammatory markers. The nuclear factor erythroid 2–related factor 2 (Nrf2)*** was assessed using ELISA technique, kit No. (E-EL-H1564), Elabscience Houston, Texas, United States. *Tumor Necrosis factor alpha (TNF alpha)* was assessed using ELISA kit NO. (E-EL-H0109), Elabscinece, Houston, Texas, United States.

***Oxidative stress markers. Glutathione peroxidase (GPX)*** was assessed using ELISA kit No. (E-EL-H5410), Elabscience, Houston, Texas, United States.

For all markers, optical density was obtained by a four-parameter logistic curve plotted on log–log graph paper and the concentration was obtained by interpolation.

#### Routine lab tests included

Liver function tests [Alanine transaminase (ALT) and Aspartate aminotransferase (AST)] and serum creatinine were also assessed.

#### Blood sampling

A 7 ml blood sample was withdrawn from each patient at baseline and after 3 months. Blood was centrifuged and part of the serum was used for analysis of the required laboratory tests and the rest was stored at – 80 °C for further analysis of GPx, TNF alpha and Nrf-2.

### Drug-related adverse effects

The occurrence and severity of drug related adverse effects were monitored in both groups and included skin rash, nausea, vomiting, diarrhea, stomach upset and constipation and were monitored regularly till the end of the study.

### Follow up

All patients were followed up every other week and were contacted weekly throughout the study period for assessment of the occurrence of any adverse drug reactions by following up on the side effect reporting card.

### End of study assessment

Included performing all the clinical tools, QOL score and laboratory assessment after the 3 months period.

### Primary outcome

Pain numerical rating scale.

### Secondary outcome

Inflammatory and oxidative stress markers (Nrf-2, GPx, TNF alpha) and QOL.

### Sample size calculation

In a previous study [1], the difference of the change in Pain numeric rating scale (NRS) between patients suffering from painful diabetic neuropathy receiving N-acetyl cysteine as adjunct therapy compared to patients receiving placebo was 1.76 with pooled standard deviation of 1.7. A minimum sample size of 21 cases in each group is required to elicit this difference at an alpha level of 0.05 and a power of the study of 90%. To guard against lost follow up, non-adherence to treatment and side effects (nearly 25%); the sample was increased to 27 cases in each group. Sample size was calculated using Power and Sample Size Calculation (PS) software version 3.1.2.

## Statistical methods

Statistical analysis was done using IBM SPSS^®^ Statistics version 26 (IBM^®^ Corp., Armonk, NY, USA). Numerical data was expressed as mean and standard deviation or median and range as appropriate. Qualitative data was expressed as frequency and percentage. Pearson’s Chi-square test or Fisher’s exact test was used to examine the relation between qualitative variables.

Comparison of quantitative variables between two groups was done using either Student t-test for normally distributed data or Mann–Whitney test (non-parametric t-test) for not normally distributed numerical data. Comparison between two consecutive measures of numerical variables was done using either paired t-test or Wilcoxon Signed Ranks test as appropriate.

Comparison between two consecutive measures of categorical variables was done using either Mc-Nemar test or Marginal homogeneity test. Correlation between numerical variables was tested using Spearman-rho correlation. Regarding multiple comparisons, p-value was corrected using Bonferroni method. All tests were two-tailed. A p-value < 0.05 was considered significant.

## Results

**Fig. 1:**
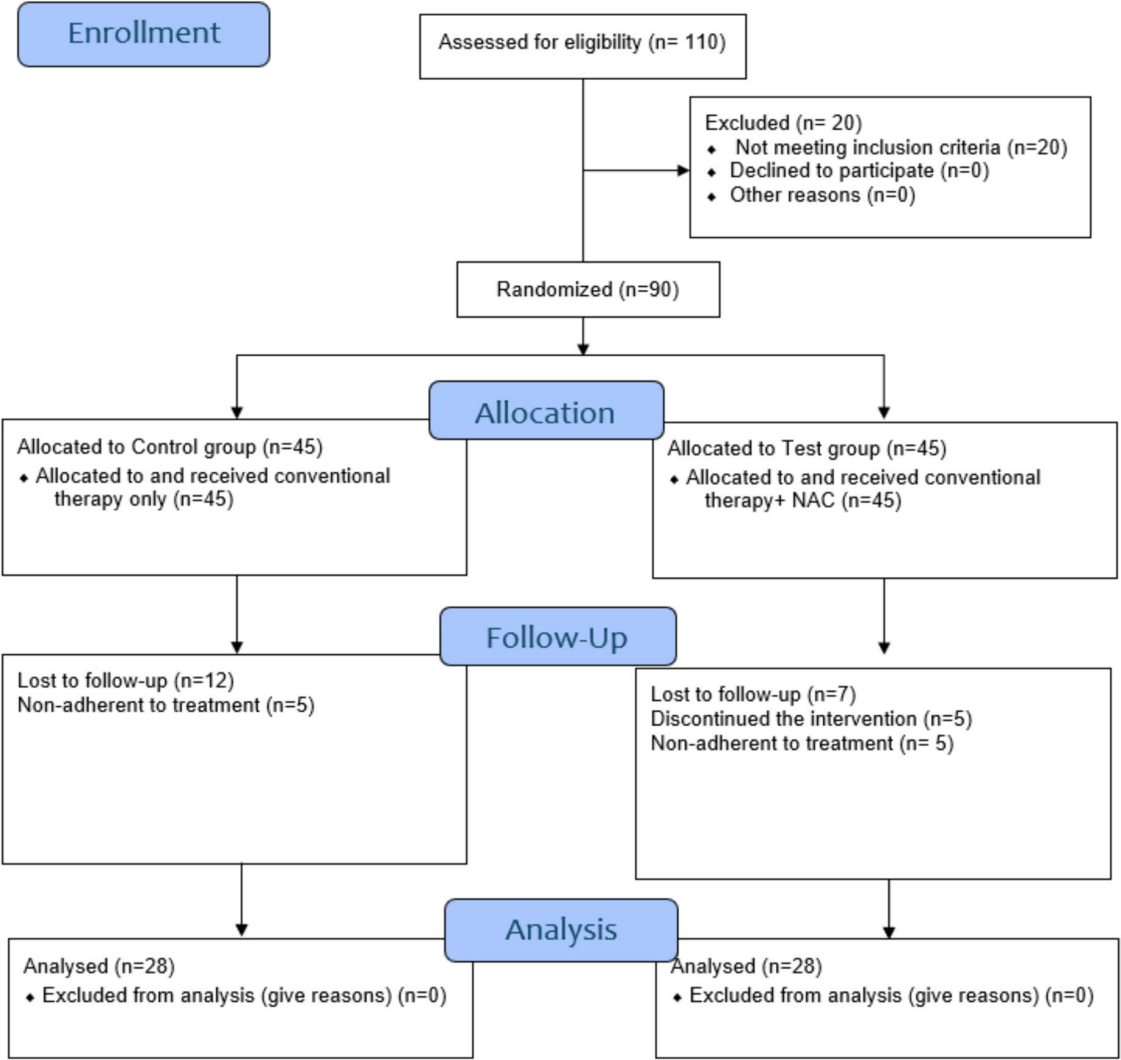
Consort flow diagram of the study

During the study period, a total of 110 diabetic patients were screened for eligibility. Only ninety patients fulfilled the inclusion criteria and were randomly assigned (by research randomizer application) into either NAC or Control group. Thirty-four were excluded from the study for various reasons. Consequently, 56 patients completed the trial period (28 patients in control group and 28 patients in intervention group). Figure 1. represents the consort flow diagram of the study.

### Demographics and patients’ histories

There were no significant differences between test and control groups regarding demographics, comorbidities, administered medications or duration of DM or DPN. Of the included patients, 7.1% (4 patients) were males and 92.9% (52 patients) were females and the gender distribution was in favor of females in both groups (85.7 and 100% in intervention and control groups, respectively). The patients’ age ranged from 38–56 years. The duration of DM ranged from (7–240) months. The demographic and clinical data of the patients are shown in Table 1.

**Table 1:** Baseline demographics & clinical characteristics

Parameter	NAC (n = 28)	Control (n = 28)	P value (between groups)
Demographics			
Age (years); mean ± S.D	47.8 ± 4.2	48.3 ± 4.4	0.642^$^
Height (cm); mean ± S.D	159.8 ± 8.6	158 ± 6.5	0.393^$^
Weight (Kg); mean ± S.D	86.5 ± 14.3	87.8 ± 14.7	0.749^$^
Waist circumference (cm): mean ± S.D	106.2 ± 11.7	109.6 ± 12.4	0.302^$^
Sex; Male; n (%) Female: n (%)	4 (14.3) 24 (85.7)	0 (0) 28 (100)	0.111^#^
Duration of illness			
Duration of DM (months); Median (range)	96 (7–240)	84 (12–192)	0.830^@^
Duration of DPN (months); Median (range)	24 (2–108)	24 (1–120)	0.545^@^
Comorbidities; n (%)			
No. of co-morbidities Hypertension Dyslipidemia Other diseases	12 (42.9) 13 (46.4) 12 (42.9) 2 (7.14)	7 (25) 15(53.6) 14 (50) 2 (7.14)	0.593^#^ 0.592^#^
Drug History; n (%)			
Anti-HTN Statin CVS protecting drugs	10 (35.7) 12 (42.9) 16 (57.1)	12 (42.9) 15 (53.6) 19 (67.9)	0.584^#^ 0.422^#^ 0.408^#^
Anti-diabetics: n (%)			
Insulin Metformin Oral drugs Oral drugs and Insulin	19 (67.9) 20 (71.4) 9 (32.1) 19 (67.9)	19 (67.9) 20 (71.4) 11 (39.3) 19 (67.9)	1^#^ 1^#^ 0.577^#^ 0.666^#^

NAC: Standard of care treatment + High dose NAC, Control: Standard of care treatment only

DM, diabetes mellitus; DPN, diabetic peripheral neuropathy; n, number; S.D, standard deviation

Statistical tests; ^#^Chi square

^@^Mann–Whitney test

^$^t-test

^*^p values < 0.05 were considered statistically significant

### Laboratory parameters

**Fig. 2:**
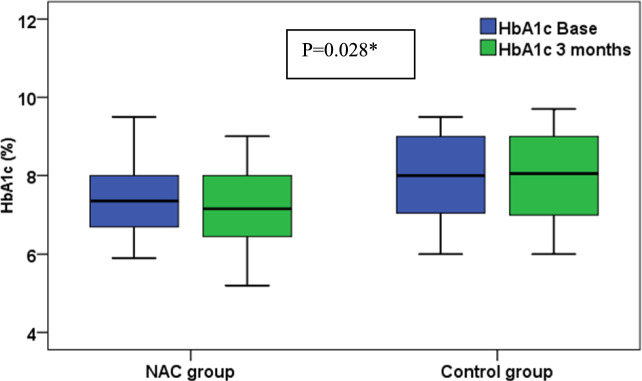
Comparison of HbA1c at baseline and after 3 months between NAC and control groups. NAC group received Standard of care treatment and High dose NAC while Control group received Standard of care treatment only

At baseline, both groups were comparable in the laboratory data namely; HbA1C, serum creatinine, AST and ALT. Also, serum creatinine and ALT were comparable between the groups at the end of the study and over time in both groups. On the other hand, HgbA1c and AST levels varied overtime and between groups. HgbA1c levels significantly declined (p = 0.02) in the NAC group versus the control that was not changed and the levels were significantly less (p = 0.028) in the NAC versus controls at the end of the study, Fig. 2. Also, AST levels showed a significant increase in the control (p = 0.004), versus a significant decline in the NAC group (p = 0.04) and NAC group levels were significantly lower than controls (29.1 vs 55.4, p = 0.012) at the end of the study, Table 2.

**Table 2:** Comparison of laboratory, inflammatory and oxidative stress markers between groups

Parameter	NAC	Control	P- value (between groups)
HgbA1C (%): mean ± S.D			
Baseline	7.4 ± 1	8.1 ± 1	0.068^$^
At the end	7.2 ± 1	8 ± 1.1	0.028^$*^
P-value (within group)	0.020^$*^	0.511^$^	0.354^@^
Percent change (%); median	− 1.95	0.00	
ALT(U/L); median (range)			
Baseline	27.9 (1.1–93.8)	11.1 (1.8–57.7)	0.104^@^
At the end	9.9 (1.7–93.8)	12.3 (2.6–61.8)	0.787^@^
P-value (within group)	0.104^^^	0.374^^^	
Percent change (%); median	− 36.99	6.53	0.017^@*^
AST(U/L); median (range)			
Baseline	49.9 (11.4–87.7)	29.4 (6.4–87.7)	0.152^@^
At the end	29.1 (9.6–87.7)	55.4 (10.8–87.7)	0.012^@*^
P-value (within group)	0.040^^*^	0.004^^*^	
Percent change (%); median	− 28.32	34.5	< 0.001^@*^
S.cr (mg/dl); median (range)			
Baseline	1.13 (0.21–7.32)	1.4 (0.13–4.21)	0.806^@^
At the end	1.15 (0.04–4.77)	1.38 (0.04–4.26)	0.922^@^
P-value (within group)	0.585^^^	0.332^^^	
Percent change (%); median	− 31.6	7.73	0.544^@^
NRF2 (pg/ml); median (range)			
Baseline	97.6 (50.9–335.7)	100.9 (63.7–4929.2) 96 (60.5–2995.3) 0.295^^^ − 9.15	0.142^@^
At the end	131.8 (63.7–425.3)	100.9 (63.7–4929.2) 96 (60.5–2995.3) 0.295^^^ − 9.15	0.364^@^
P-value (within group)	< 0.001^^*^	100.9 (63.7–4929.2) 96 (60.5–2995.3) 0.295^^^ − 9.15	
Percent change (%); median	25.3	100.9 (63.7–4929.2) 96 (60.5–2995.3) 0.295^^^ − 9.15	0.002^@*^
TNF (pg/ml); median (range)			
Baseline	53.1 (7.7–243.1)	11.6 (7.4–133.2)	0.364^@^
At the end	9.3 (7.4–134.3)	18.1 (8.3–132.9)	0.364^@^
P-value (within group)	< 0.001^^*^	0.02^^*^	
Percent change (%); median	− 21.45	4.9	< 0.001^@*^
GPx (pg/ml); median (range)			
Baseline	161.2 (18.4–321.4)	185.2 (11.4–583.7)	0.306^@^
At the end	346.5 (73.3–967.9)	153.3 (6.8–491.5)	< 0.001^@*^
P-value (within group)	< 0.001^^*^	0.012^^*^	
Percent change (%); median	100	22.75	< 0.001^@*^

NAC: Standard of care treatment + High dose NAC, Control: Standard of care treatment only NRF2: Higher NRF2 levels indicates reduced oxidative stress and inflammation [1].TNF: Higher TNF levels indicate higher inflammation and increased DPN severity [2]. GPx: Higher GPx levels indicate higher anti-oxidant effect and improved DPN symptoms [3]

NRF2*:* The nuclear factor erythroid 2–related factor 2; TNF: tumor necrosis factor alpha; GPx: Glutathione peroxidase; S.D: standard deviation; HgbA1C: Glycated hemoglobin; ALT: Alanine transaminase; AST: aspartate aminotransferase; S.cr: serum creatinine

Statistical tests; ^@^ Mann–Whitney test

^^^Wilcoxon signed ranks tests

^$^t-test

^*^p values < 0.05 were considered statistically significant

**Fig. 3:**
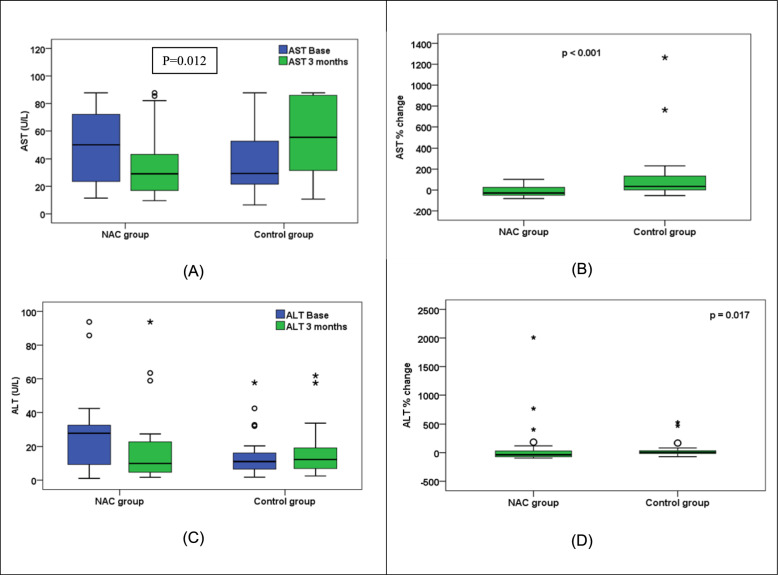
Comparison of liver enzymes between NAC and control groups at baseline and after 3 months (**A**) AST levels (**B**) AST percent change (**C**) ALT levels (**D**) ALT percent change. NAC group received Standard of care treatment and High dose NAC while Control group received Standard of care treatment only.

As for the percent change overtime, it was decreased by 28.32% in the NAC group versus controls that increased by 34.5% (p = 0.001), Fig. 3. Although ALT didn’t show significant change between NAC and controls, there was a significant percent change overtime decreasing by 36.99% in NAC group versus controls that increased by 6.53% (P = 0.017), Fig. 3.

### Clinical scores

**Fig. 4:**
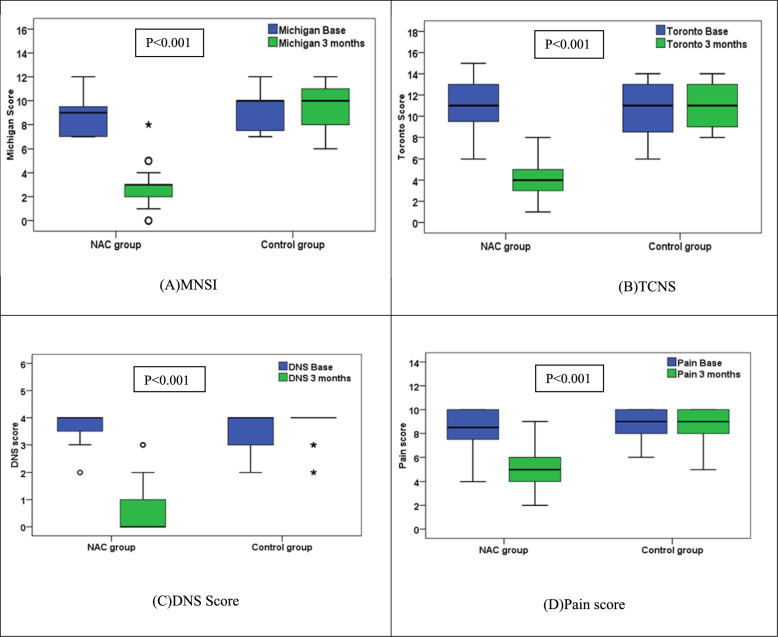
Comparison of clinical scores at baseline and after 3 months between NAC and control groups, (**A**) Michigan score (**B**) Toronto score s (**C**) DNS score (**D**) Pain score. NAC group received Standard of care treatment and High dose NAC while Control group received Standard of care treatment only

At baseline, both groups were comparable in all the clinical scores; MNSI, TCNS, DNS and Pain scores. After 3 months of treatment, there were a significant change in all the score between the 2 groups. All the scores, MNSI, TCNS, DNS and pain scores significantly decreased overtime in the NAC group (p < 0.001) while it was not changed in the control group. After the 3 months of NAC administration, the NAC group median score was significantly lower than the control group in the MNSI score (3 vs 10, p < 0.001), the TCNS (4 vs 11, p < 0.001), the DNS (0 vs 4, p < 0.001) and the pain score (5 vs 9, p < 0.001) respectively, Table 3, Fig. 4.

**Table 3:** Comparison of Clinical scores between groups at baseline and 3-months after treatment

Parameter	NAC	Control	P-value (between groups)
MNSI; median (range)			
Baseline	9 (7–12)	10 (7–12)	0.140 < 0.001^@*^
At the end	3 (0–8)	10 (6–12)	0.140 < 0.001^@*^
P-value (within group)	< 0.001^^*^	0.340^^^	
TCNS; Median (Range)			
Baseline	11 (6–15)	11 (6–14)	0.829
At the end	4 (1–8)	11 (8–14)	< 0.001^@*^
P-value (within group)	< 0.001^^*^	0.248^^^	
DNS; median (range)			
Baseline	4 (2–4)	4 (2–4)	0.719 < 0.001^@*^
At the end	0 (0–3)	4 (2–4)	0.719 < 0.001^@*^
P-value (within group)	< 0.001^^*^	0.157^^^	
Pain score; median (range)			
Baseline	8.5 (4–10)	9 (6–10)	0.368
At the end	5 (2–9)	9 (5–10)	< 0.001^@*^
P-value (within group)	< 0.001^^*^	0.785^	

NAC: Standard of care treatment + High dose NAC, Control: Standard of care treatment only

MNSI, TCNS, DNS and Pain score: The lower the score denotes an improvement in symptoms while the higher the score denotes worsening of symptoms [3, 4]

MNSI: Michigan neuropathy screening instrument; TCNS: Toronto clinical neuropathy score; DNS: Diabetic neuropathy score

Statistical tests; ^@^Mann–Whitney test

^^^Wilcoxon signed ranks tests

^*^p values < 0.05 were considered statistically significant

### Inflammatory and oxidative stress markers

At baseline, both groups were comparable in NRF2, TNFα and GPX serum levels, Table 3. After 3 months of treatment, levels of NRF2, TNFα and GPx significantly differed between the 2 groups, Table 2.

**Fig. 5:**
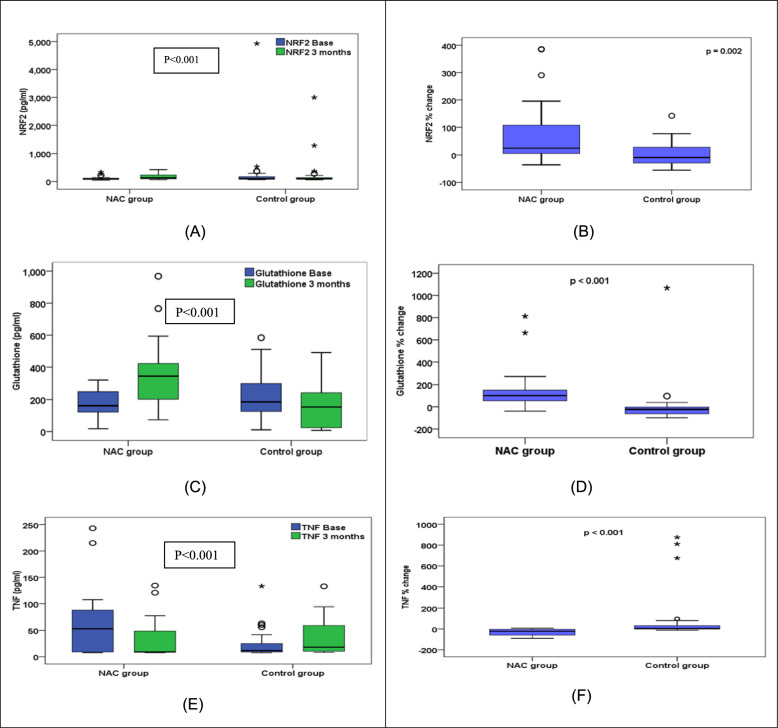
Comparison of NRF2, GPx and TNF alpha between NAC and control groups at baseline and after 3 months (**A**) NRF2 levels (**B**) NRF2 percent change (**C**) GPx levels (**D**) GPx percent change (**E**) TNF-a levels (**F**) TNF-a percent change. NAC group received Standard of care treatment and High dose NAC while Control group received Standard of care treatment only

NRF2 levels significantly increased in the NAC group (p < 0.001) versus control that declined overtime. The percent change overtime was increased by 25.3% in the NAC group versus controls that decreased by 9.15% (p < 0.001), Fig. 5.

Also, the GPX levels significantly increased in the NAC group (p < 0.001) versus a significant decline in the control (p = 0.012) and the NAC group levels were significantly higher than controls (346.5 vs 153.3, p < 0.001) at the end of the study. The percent change overtime was increased by 100% in the NAC group versus controls that decreased by 22.75% (p < 0.001), Fig. 5.

On the contrary, TNFα levels significantly declined in the NAC group (53.1 vs 9.3, p < 0.001) versus the control group that increased (11.6 vs 18.1, p = 0.02). The percent change overtime was decreased by 21.45% in the NAC group versus controls that increased by 4.9% (p < 0.001), Fig. 5.

### Quality of life evaluation

When evaluating the quality-of-life domains, both groups were comparable at baseline. After 3 months of treatment, there were a significant change between the groups in the overall QOL score as well the individual scores for anxiety, energy and mobility.

**Fig. 6:**
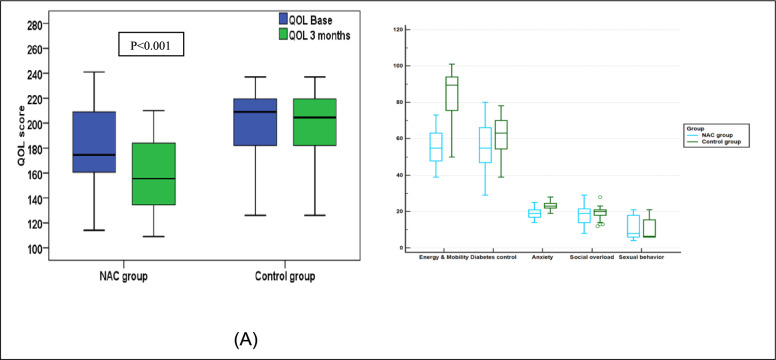
Comparison of Total and individual domains of QOL scores at baseline and after 3 months between NAC and control groups. **A** Total QOL score (**B**) Individual QOL domains. NAC group received Standard of care treatment and High dose NAC while Control group received Standard of care treatment only

After 3 months of treatment with N-acetyl cysteine, the NAC group reported a significant decrease in the total QOL score and the individual QOL scores for anxiety and energy and mobility (< 0.001) versus the control group that showed no change. At the end of the study, the NAC group scores were significantly lower than the control group in total QOL score and the individual QOL scores for anxiety and energy and mobility (P < 0.001), Table 4, Fig. 6.

**Table 4:** Quality of life domains of both groups at baseline and 3-months after treatment

Parameter	NAC	Control	P-value (between groups)
Energy and mobility; median (range)			
Baseline	76 (43–102)	90 (50–101)	0.092^@^
At the end	55 (39–73)	89.5 (50–101)	< 0.001^@*^
P-value (within group)	< 0.001^^*^	0.066^^^	
Diabetes control; median (range)			
Baseline	55 (29–80)	63 (39–78)	0.106^@^
At the end	55 (29–80)	63 (39–78)	0.106^@^
P-value (within group)	0.317^^^	1^^^	
Anxiety; median (range)			
Baseline	22 (16–27)	23 (19–28)	0.060^@^
At the end	19 (14–25)	23 (19–28)	< 0.001^@*^
P-value (within group)	< 0.001^^*^	0.152^^^	
Social overload; median (range)			
Baseline	19 (8–29)	20 (12–28)	0.396^@^
At the end	19 (8–29)	20 (12–28)	0.396^@^
P-value (within group)	1^^^	1^^^	
Sexual behavior; median (range)			
Baseline	8 (4–21)	6.5 (6–21)	0.960^@^
At the end	8 (4–21)	6.5 (6–21)	0.960^@^
P-value (within group)	1^^^	1^^^	
QOL total; median (range)			
Baseline	174.5 (114–241)	209 (126–237)	0.188^@^
At the end	155.5 (109–210)	204.5 (126–237)	< 0.001^@*^
P-value (within group)	< 0.001^^*^	0.108^^^	

NAC: Standard of care treatment + High dose NAC, Control: Standard of care treatment only QOL total and its domains: The higher the value denotes a worsening quality of life and the lower the value denotes a better quality of life [5]

QOL: Quality of life

Statistical tests; @ Mann–Whitney test

^Wilcoxon signed ranks tests

^*^p values < 0.05 were considered statistically significant

### Drug-related adverse effects

Only 3 patients in the NAC group suffered skin rash that was mild and did not require therapy discontinuation and spontaneously resolved on the next day. Two patients reported nausea, on drug start that was mild and did not affect their adherence. Five patients in the NAC group experienced side effects as nausea, vomiting and stomach upset and despite being mild symptoms they refused to continue treatment and were excluded from the analysis.

### Correlations

The MNSI score was found to be highly positively correlated with TCNS (P < 0.001, r = 0.872) and DNS (P < 0.001, r = 0.841) and moderately to the D-39 QOL score (P < 0.001, r = 0.581). Also, the TCNS was highly positively correlated with DNS (P < 0.001, r = 0.875) and moderately with the D-39 QOL score (P < 0.001, r = 0.556). GPx was negatively correlated to MNSI (P < 0.001, r = − 0.450), TCNS (P < 0.001, r = − 0.588), DNS (P = 0.001, r = − 0.441) and D-39 QOL score (P = 0.029, r = − 0.291). And the D-39 QOL score was positively correlated to DNS (P < 0.001, r = 0.64) and pain score (P < 0.001, r = 0.552).

## Discussion

To the best of our knowledge, this study is the first study to assess the effect of high dose N-acetyl-cysteine (NAC) (2400 mg/day) as an adjuvant therapy on the clinical outcomes, inflammatory and oxidative stress markers and QOL of diabetic peripheral neuropathy patients.

Results of the current study showed that high dose NAC, improved the inflammatory marker NRF-2 by 25.3% and the oxidative stress marker GPx by 100% and resulted in a decline in the TNF alpha levels by 21.45%. Also, NAC administration resulted in an improvement in the DPN scores and an alleviation in painful symptoms assessed by pain scores which improved by 40% together with a marked improvement in patient’s quality of life versus controls with improvement by 10.9% in NAC versus controls.

NAC as an antioxidant, aids in the synthesis of glutathione (GSH) and replenishes the GSH pool that diminishes due to oxidative stress and inflammation. NAC has the ability to scavenge free radicals in vitro and in vivo [32] Antioxidant and anti-inflammatory properties of NAC are more pronounced at higher doses. Supplementing with N-acetyl cysteine raises glutathione levels, the and inhibits the production of inflammatory cytokines such as TNF-alpha and interleukin-8 [33]

NAC administered at doses reaching as high as 8000 mg/day, were not associated with any clinically significant adverse events. Few reports of severe and occasionally fatal anaphylactoid reactions were reported with intravenous administration of the supplement at high doses. There has only been a few mild gastrointestinal disturbances like heartburn, nausea, and vomiting linked to oral NAC therapy. Oral NAC is therefore a compelling therapeutic option in models of inflammatory and neuropathic pain due to its outstanding efficacy and high margin of safety [34]

In the current study, only few patients experienced mild side effects like skin rash and nausea that did not require NAC discontinuation. Some of the patients who reported mild symptoms of nausea, vomiting and stomach upset requested discontinuing the medication and were withdrawn from the study.

Reduced cellular function brought on by oxidative stress, results in cellular death and free radical-induced liver necrosis. Up until the time of significant liver damage, liver function tests can be used to track this reduced cellular function—they might even be the only indicator. Supplemental antioxidants protect cells from this oxidative damage [35].

In the current study, NAC administration resulted in a significant decrease in AST levels in the NAC group versus controls, indicating a protective effect of NAC administration on oxidative stress-induced liver injury and an improvement in antioxidant levels. Similarly, Khoshbaten et al. in their study assessing liver functions in patients with Non- alcoholic fatty liver disease, a significant decrease in ALT levels was reported after NAC supplementation [35]

*Glycated hemoglobin* measurement reflects a mean glucose concentration over the preceding 1–2 months, in contrast to a one-time glucose level. Panahi et al., stated in their study that NAC consumption diminished fasting plasma glucose (P = 0.02), fasting serum insulin (P = 0.006) and insulin resistance index (P = 0.005) indicating an overall improvement in glycemic homeostasis[36]. This was also previously proven in a previous in vivo study on diabetic rats [37]. Similarly, in the current study, after 3 months, the NAC-treated group reported a significant decline in HgbA1C versus controls. On the contrary, Szkudlinska et al. reported no change in glycemic control, glucose tolerance or insulin release with either doses of NAC 600 mg and 1200 mg twice daily [38] This discrepancy maybe attributed to the difference in timing of sample withdrawal relative to NAC administration between the different studies.

NAC effect on glycemic control was proposed to be due to a reduction in Monocyte chemoattractant protein-1 (MCP-1) levels, which has a vital role in inflammation where it enhances the expression of other inflammatory factors, [39] increases insulin sensitivity and thereby improves glucose metabolism. Circulating MCP-1 and CRP levels are elevated in insulin resistant states such as obesity, impaired glucose tolerance, and type 1 and 2 diabetes [37]

According to Oh et al., *Michigan neuropathy screening instrument* is an effective tool for screening diabetic peripheral neuropathy [48] Several studies have used MNSI in screening for DPN before and after treatment. The present study is the first to assess MNSI before and after treatment with N- acetylcysteine. According to Didangelos et al., MNSI was not significantly reduced after vitamin b-12 administration as an anti-oxidant intervention in DPN [49]. Whereas, the present study showed a significant decline in MNSI only in the NAC-treated group, while the control group showed no change. In line with our findings, another study by Boghdadi et al., compared vitamin B complex combined with Alpha Lipoic Acid versus vitamin B complex alone in treatment of Diabetic Polyneuropathy Type 2 diabetic patients and reported a significant decrease in MNSI in the alpha lipoic acid group [50] These findings highlight the impact of powerful antioxidants and their effect on neuropathy outcomes reflected in the MNSI.

N-acetyl cysteine was previously reported to improve neuropathic symptoms and as a consequence it decreased MNSI [22]

*Toronto clinical neuropathy score* was validated for the diagnosis and staging of a wide spectrum of polyneuropathies [51]. No clinical trials till today have used TCNS for assessing interventions in DPN, but only one study assessed life style interventions in diabetic peripheral neuropathy using TCNS [52] and reported a favorable decrease in the score with life style interventions. Similarly, the current study reported a significant decline in the TCNS only in the NAC-treated group, indicating the beneficial effects of NAC on improving the severity of DPN and ameliorating the nerve injury.

Our present study also used diabetic neuropathy symptom score as one of the tools to diagnose and screen for diabetic peripheral neuropathy [28].

Pain score and D-39 quality of life questionnaire were used to assess severity of pain and improvement in quality of life. The present study showed a significant decrease (p < 0.001) in both scores in the NAC-treated group, while the control did not show any significant change. The overall QOL was significantly improved in the NAC-treated group together with a positive improvement in the domains pertaining to anxiety and energy and mobility. These findings elaborate the effect of NAC on improving neuropathy that is directly reflected on improving patients’ mobility, energy level and anxiety. Similarly, Heidari et al., showed that pain score was significantly decreased when comparing a group taking NAC with the placebo group [1] and which contributed to improving the overall quality of life.

*NRF2* is a protein that has a prominent role in controlling the antioxidant defense system's reaction to insults like oxidative stress-related stimuli [3]. It is believed that the Nrf2 signaling pathway has a protective effect in inflammatory diseases [40]. Hence, increasing NRF2 indicates stimulating pathways that resolve inflammation by reducing oxidative stress, inhibiting pro-inflammatory signaling and restoring the function of mitochondria.

The present study showed a significant increase in NRF2 in the NAC group after 3 months of treatment with NAC (P < 0.001) as compared to the control that showed a decline in NRF2 levels. To the best of our knowledge, till today no clinical studies have assessed the effect of NAC on NRF2 levels in diabetic neuropathy. In line with our findings, Jannatifar et al., reported a significant increase in NRF2 levels (p = 0.01) with NAC administration, in their study evaluating the effect of NAC on NRF2 in men with Asthenoteratozoospermia [41]. Similarly, Yamamoto et al., mentioned that NAC administration elevated the NRF2 expression levels in the lung in a mouse model exposed to phosgene, even 3 h after exposure. Revealing that upregulating the NRF2/glutathione reductase pathway may result in the synthesis of reduced glutathione, which would mediate the preventative action of NAC [42].

*GPx* is one of the main antioxidants acting as the first line of defense against free radical damage, essential for maintaining optimum health and well-being and is highly correlated to neuropathy [43, 44]. Increasing GPx levels is essential for halting oxidative stress and alleviating painful symptoms.The current study reported a significant increase in GPx levels from baseline to end in the NAC group versus controls that reported a decline in GPx levels overtime. At 3 months, the NAC group GPx levels were significantly higher versus controls (P < 0.001). Similarly, Heidari et al., in their study evaluating the ameliorative effects of NAC as adjunct therapy on symptoms of painful diabetic neuropathy, the serum level of GPx was significantly higher in groups receiving NAC compared to controls (P < 0.001) [1]. Also, Panhi et al., in their study assessing the effect of acetyl cysteine on metabolic status in patients with metabolic syndrome, confirmed that consumption of NAC at a dose of 1800 mg/day for 12 weeks enhanced plasma GSH levels (p < 0.001) [36]. On the other hand, Szkudlinska et al., reported no change in GPx levels in neither the group taking 600 mg bid or the group taking 1200 mg bid, and they attributed this to the fact that reactive oxygen species have very short half-lives and are challenging to be measured directly and that oxidative stress byproducts are quantified using surrogate indicators of oxidative stress [38]. The discrepancy between this study and the current study could be attributed to differences in sample sizes, study duration, analysis methodologies and various patients’ characteristics.

*TNF*-alpha, the proinflammatory cytokine, is implicated in the beginning and persistence of inflammatory cascades that lead to nerve injury, demyelination, and decreased nerve conduction [45]. Hence elevated TNF alpha levels result in magnification and potentiation of excess inflammation, which in turn leads to increased pain and nerve injury.

The present study showed a significant decline in TNF-alpha levels overtime in the NAC group compared to a significant increase overtime in the control group (P < 0.001). At 3 months, TNF-alpha levels were significantly higher in the NAC versus control group. Till today, no clinical trials have addressed the effect of NAC on TNF levels in diabetic neuropathy. Similar to our findings, Zhang et al., in their study evaluating NAC effect on oxidative and inflammatory response in patients with community acquired pneumonia, reported a decline in TNF-alpha levels in the NAC group versus the non- NAC group (P < 0.05) [46] On the other hand, a study by Oner and Muderris reported an increase in TNF-alpha in women with polycystic ovary syndrome before and after taking acetyl cysteine. [47]. The differences between studies could be attributed to differences between the two disease states and patient population.

The current study also showed a significant negative correlation between the Michigan neuropathy screening instrument and glutathione peroxidase (P < 0.001, r = − 0.450) while a positive correlation between Michigan neuropathy screening instrument and tumor necrosis factor (P = 0.048, r = 0.266), indicating the impact of halting inflammation and oxidative stress on improving the patients’ overall neuropathy severity. Similarly, according to Buyukaydin et al., in his study on the relationship between diabetic polyneuropathy, serum visfatin and oxidative stress biomarkers, he reported a positive linear relationship between MNSI, total oxidant status and oxidative stress index [53]. Moreover, Etienne et al., in analyzing oxidative stress markers measured by malondialdehyde reported a decreased total antioxidant capacity in the serum of patients with type 2 DM and DPN, when compared with normal participants [54].

According to Malazy et al. in his study on the prevalence of diabetic peripheral neuropathy and related factors, the authors reported that the strongest linear correlation was between MNSI and DNS (r = 0.7) [55], coinciding with the strong positive correlation between MNSI and DNS (P < 0.001, r = 0.841) that was reported in the present study.

Sif et al., in his study in diabetic polyneuropathy and pain, prevalence, and patient characteristics stated that there was a negative correlation between the reported QOL and the intensity of pain (Spearman's rho − 0.24, P < 001) and a negative correlation between QOL and DPN, which is in line with the present study that showed a positive significant correlation between QOL score and pain score (P < 0.001, r = 0.552), indicating a decline in patients’ QOL with pain. In the current study, we measured QOL using D-39 questionnaire, with a score of 100 denoting an impaired QOL while zero indicates the best QOL, hence explains the negative correlation between QOL and pain [56].

Despite the novelty of the current study regarding the effect of high dose NAC, it bears the limitations of a relatively small sample size and the inability to use placebo.

It is hence recommended to conduct larger multi-center studies to confirm the findings of the current study.

In conclusion, high dose NAC administration ameliorated inflammation and oxidative stress by increasing GPx and NRF2 and decreasing TNF-alpha. NAC also improved the clinical outcomes of DPN by improving MNSI, TCNS, DNS and pain score and enhancing QOL with a favorable safety profile and minimal side effects. High dose NAC is a promising candidate for use as adjunctive therapy in DPN patients to improve DPN outcomes.

## Recommendations

We recommend conducting larger multicenter RCT to confirm the results of the current study. Also studying the effect of different NAC doses for longer periods.

## Data Availability

No datasets were generated or analysed during the current study.
